# Continued Growth of the Impact Factors of MDPI Open Access Journals

**DOI:** 10.3390/molecules15064450

**Published:** 2010-06-21

**Authors:** Dietrich Rordorf

**Affiliations:** MDPI AG, Postfach, CH – 4005 Basel, Switzerland; Office: Kandererstrasse 25, CH – 4057 Basel, Switzerland; Tel. +41 61 683 77 35; Fax: +41 61 302 8918; E-Mail: rordorf@mdpi.com

We are pleased to report the continued increase of the Impact Factors of MDPI journals in 2009 (see Table 1 and Figure 1). 

As noted in a similar Editorial published in June 2009, the Impact Factors of MDPI journals have been steadily recovering since the inception of the full Open Access publishing policy in early 2007 [1]. We are pleased to see that all journals now have an Impact Factor much higher than 1. In 2011 the Journal Citation Report (JCR) will include the first official Impact Factor of *Entropy*, and in 2012, the *International Journal of Environmental Research and Public Health* will be included for the first time. Nevertheless, we are already able to compute a preliminary impact factor of approximately 1.4 for *Entropy* [2].

## Figures and Tables

**Figure 1:**
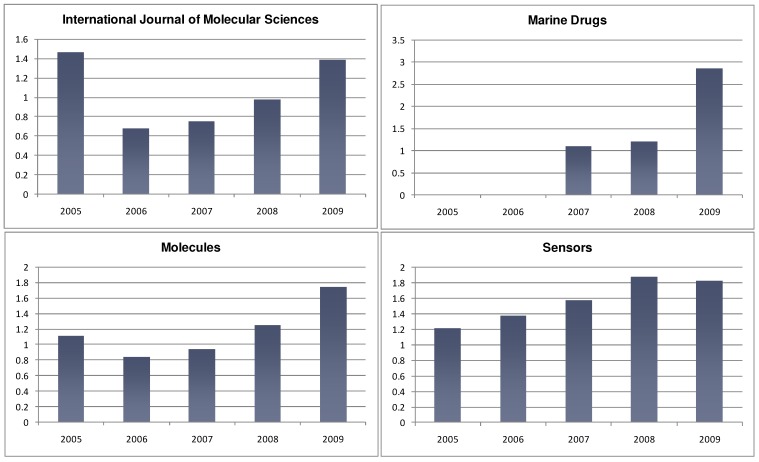
Impact Factor Progress 2005-2009

**Table 1:** Impact Factors of four MDPI journals (adapted from the Journal Citation Report (JCR), Edition 2009, Copyright 2010 by Thomson Reuters).

Journal	2005	2006	2007	2008	2009
**IJMS**	1.467	0.679	0.750	0.978	1.387
**Marine Drugs**	n/a	n/a	1.103	1.200	2.863
**Molecules**	1.113	0.841	0.940	1.252	1.738
**Sensors**	1.208	1.373	1.573	1.870	1.821
